# Strigolactones Improve Plant Growth, Photosynthesis, and Alleviate Oxidative Stress under Salinity in Rapeseed (*Brassica napus* L.) by Regulating Gene Expression

**DOI:** 10.3389/fpls.2017.01671

**Published:** 2017-09-27

**Authors:** Ni Ma, Chao Hu, Lin Wan, Qiong Hu, Junlan Xiong, Chunlei Zhang

**Affiliations:** ^1^Oil Crops Research Institute, Chinese Academy of Agricultural Sciences, Wuhan, China; ^2^Key Laboratory of Oil Crop Biology, Ministry of Agriculture, Wuhan, China; ^3^College of Plant Science and Technology, Huazhong Agricultural University, Wuhan, China

**Keywords:** winter rapeseed (*B. napus* L.), strigolactones, salinity stress, photosynthesis, RNA-sequencing (RNA-Seq)

## Abstract

Rapeseed (*Brassica napus* L.) is a very important edible oil crop in the world, and the production is inhibited by abiotic stresses, such as salinity. Plant hormones can alleviate the stress by regulating the physiological processes and gene expression. To study the plant responses to salinity in combination with GR24, a synthesized strigolactone, the oilseed rape variety (Zhongshuang 11) replications were grown in the pots in a controlled growth chamber under three levels of salinity (0, 100, and 200 mM NaCl) and 0.18 μM GR24 treatments at the seedling stage for 7 days. The results showed that salinity depressed the shoots and roots growth, whereas GR24 improved the growth under salt stress. Leaf chlorophyll contents and gas exchange parameters (net photosynthetic rates, stomatal conductance, intercellular CO_2_ concentration, and transpiration rate) were also reduced significantly with increasing salinity, and these effects could be partially reversed by GR24 application. Additionally, GR24 treatment significantly increased and decreased the photosystem II quantum yield and non-photochemical quenching, respectively, under salinity stress conditions. The activities of peroxidase and superoxide dismutase increased, and lipid peroxidation measured by the level of malondialdehyde reduced due to GR24 application. The transcriptome analysis of root and shoot was conducted. Three hundred and forty-two common differentially expressed genes (DEGs) after GR24 treatment and 166 special DEGs after GR24 treatment under salinity stress were identified in root and shoot. The DEGs in root were significantly more than that in shoot. Quantitative PCR validated that the stress alleviation was mainly related to the gene expression of tryptophan metabolism, plant hormone signal transduction, and photosynthesis.

## Introduction

Salinity is recognized as one of the major abiotic stresses that limits plant growth and productivity, particularly in arid and semi-arid climates (Yamaguchi and Blumwald, 2005; Rozema and Flowers, 2008). More than 800 million hectares of arable lands globally are adversely affected by salinity (Munns and Tester, 2008), which is equivalent to approximately 20% of the world’s cultivated land area and 50% of all irrigated lands (Rhoades and Loveday, 1990; Sairam and Tyagi, 2004).

Salinity initiates complex responses to inhibit plant growth and physiological processes (Munns et al., 2006; Albacete et al., 2008; Naeem et al., 2012). It is reported that salinity stress mainly limits photosynthesis and CO_2_ diffusion through a decrease of stomatal and the mesophyll conductances (Flexas et al., 2004). Acosta-Motos et al. (2015) also mentioned that the negative impact of salinity stress on the photosynthetic apparatus, especially photosystem II (PSII), could be detected by imaging chlorophyll fluorescence, a non-invasive and quantitative tool (Buschmann et al., 2000; Oxborough, 2004). In addition to these stress effects, oxidative stress may also occur.

Salinity stress generates oxidative damage through the production of reactive oxygen species (ROS) including hydrogen peroxide (H_2_O_2_) among others (Munns and Tester, 2008). The stressed plants can synthesize antioxidants in cells such as superoxide dismutase (SOD) and a variety of peroxidases (POD) as ROS scavengers (Wang et al., 2014). Malondialdehyde (MDA) as the decomposition product of polyunsaturated fatty acids of biomembranes showed greater accumulation under salinity stress (Jan et al., 2017).

Plant hormones are involved in reconfiguration of developmental patterns in response to abiotic stress (Zhou and Leul, 1998; Peleg and Blumwald, 2011; Zwack and Rashotte, 2015). Strigolactones (SLs) are a new group of putatively carotenoid-derived terpenoid lactones isolated from root exudates which can stimulate the seed germination of the root parasitic plant *Striga* (Cook et al., 1972; Yoneyama et al., 2008). These new group of phytohormones have been suggested to play a pivotal role in the regulation of above ground plant architecture and root development (Koltai et al., 2010; Brewer et al., 2013; Foo and Reid, 2013; Cuyper et al., 2015). GR24, a synthesized strigolactone, is involved in response to abiotic stress. As a positive regulator in stress response, exogenous GR24 can enhance the drought and salt tolerance of *Arabidopsis* (Ha et al., 2014; Kapulnik and Koltai, 2014). A comparative microarray analysis of the leaves of the SL-response max2 mutant and WT plants under normal and dehydrative conditions revealed multiple hormone-response pathways controlling the adaptation to environmental stress (Ha et al., 2014). RNA-sequencing (RNA-Seq) technology is a cost-effective and high-throughput platform for transcriptome analysis that has efficiently and economically investigated various gene expressions, even for numerous non-model species (Guo et al., 2010; Wang et al., 2010). Nevertheless, little information has been available to elucidate the effects of SLs treatment on the photosynthesis, oxidative stress, and transcriptomics in plants under salinity stress.

Oilseed rape (*Brassica napus* L.) is one of the world’s major oilseed crops (Diepenbrock, 2000) and the most important source of edible oil in China (Momoh and Zhou, 2001). As the growth of rapeseed is threatened by salinity stress, it is imperative to find approaches to improve the vigorousness under this abiotic stress. In our previous work, we found that the root and shoot growth of rapeseed were improved after treated with appropriate concentration of GR24 for 7 days (unpublished data). Their responses to GR24 under saline conditions are almost unknown. Thus, the objectives of the present investigation were (1) to identify the interaction of salinity and GR24 on leaf photosynthetic capacity and chlorophyll fluorescence, and (2) to elucidate the transcriptome information. All these will provide important insight into plant growth under abiotic stress and the responses to hormonal stimuli.

## Materials and Methods

### Plant Materials and Growth Conditions

Zhongshuang 11 (ZS 11), an elite conventional cultivar, was sown in the plastic pots (120 mm × 150 mm) filled with soil. Fifteen days after sowing, morphologically uniform seedlings were retained with one plant per pot containing a modified half-strength Hoagland nutrient solution (Arnon and Hoagland, 1940) with some modification in the greenhouse. Approximately 360 pots were prepared. The solution was renewed every 5 days. The seedlings were grown under light intensity in the range of 250–350 μmol m^-2^ s^-1^. The temperature was in the range of 22–25°C, and the relative humidity was approximately 55–60%.

After a 2-week acclimatization period, the pots were separated to two groups. In one group, 180 pots were treated with desired salinities (0, 100, and 200 mM NaCl) using 60 pots per treatment. In the other group, 180 pots were simultaneously treated with an aqueous solution of GR24 at a concentration of 0.18 μM combined with 0, 100, and 200 mM NaCl. Plants treated with distilled water were served as the mock. Preliminary studies were carried out to determine the morphology and physiological characteristics. Seven days after treatments, data for biomass of root and shoot (fresh and dry), photosynthetic gas exchange parameters, and chlorophyll fluorescence were recorded. The chlorophyll content, lipid peroxidation assay, and activities of antioxidant enzymes were measured as well.

The second experiment was conducted by using 60 pots. The plants were cultivated as above. Before GR24 and NaCl treatment, the roots and leaves of nine seedlings were sampled and separated for three replicates, respectively. At 12 h of 0.18 μM GR24 in combined with salt treatments (0 and 200 mM NaCl), seedling roots and leaves of four treatments were sampled with three replicates by mixing three seedlings per replicate, respectively. Shoots and roots were collected and frozen immediately in liquid nitrogen for RNA-Seq.

### Determination of Plant Biomass

Dry and fresh weights of shoots and roots were measured separately 7 days after treatment. Fresh shoots and roots of 10 plants per treatment with three replicates were weighed immediately after harvesting and then placed in the oven at 80°C to constant weight (Momoh and Zhou, 2001).

### Chlorophyll Content

Chlorophyll content was measured as soil and plant analyzer development (SPAD) value on the intact topmost fully expanded leaf using a chlorophyll meter (Minolta, Japan) which provides a rapid, accurate, and non-destructive estimate of leaf chlorophyll content. Six data per treatment were collected with three replicates (Wu et al., 1998).

### Photosynthetic Parameters

Gas exchange analysis was conducted using the Portable Photosynthesis System (LI-6400, United States) on the leaves between 9 AM and 11 AM. The net photosynthetic rates (Pn), the stomatal conductance (Gs), intercellular CO_2_ concentration (Ci), and transpiration rate (Tr) were detected. The CO_2_ concentration of the leaf chamber was 400 μmol mol^-1^. The air flow speed was 500 μmol s^-1^. The photosynthetically active radiation (PAR) was 1,000 μmol m^-2^ s^-1^. The leaf temperature was 25 ± 1.5°C, and the air relative humidity was 80–90%. The data were automatically collected every 2–3 min with at least six replicates.

### Chlorophyll Fluorescence

A pulse-amplitude modulated photosynthesis yield analyzer (Image-PAM; Walz GmbH, Effeltrich, Germany) was used to measure chlorophyll fluorescence. The leaves were dark adapted for 30 min prior to determine measure the chlorophyll fluorescence parameters. Data were automatically collected at six leaves per treatment. Three replicates for each treatment were adopted. The parameters were determined according to the publication by Acosta-Motos et al. (2015).

### Lipid Peroxidation Assay

The level of lipid peroxidation, MDA content was determined. Plant fresh tissues (0.5 g) were homogenized and extracted in 10 ml of 0.25% TBA made in 10% trichloroacetic acid (TCA). The extract was heated at 95°C for 15 min and then quickly cooled on ice. After centrifugation at 5,000 × *g* for 10 min, the absorbance of the supernatant was measured at 532 nm. Correction of non-specific turbidity was made by subtracting the absorbance value taken at 600 nm. The level of lipid peroxidation was expressed as nanomoles per gram fresh weight by using an extinction coefficient of 155 mM cm^-1^.

### Activity of Antioxidant Enzymes

Antioxidants enzymes (guaiacol POD and SOD) activity was determined according to Zhang (1992) with some modifications. Leaf samples were ground with a mortar and pestle under the chilled condition in the homogenization buffer. The homogenate was centrifuged at 10,000 × *g* for 20 min at 4°C, and the supernatants were used for enzyme assays.

Guaiacol POD activity was measured with guaiacol as the substrate in a total volume of 3 ml. The reaction mixture consisted of 50 mM potassium phosphate buffer (pH 6.1), 1% guaiacol, 0.4% H_2_O_2_, and enzyme extract. Increase in the absorbance due to oxidation of guaiacol was measured at 470 nm.

Superoxide dismutase activity was assayed by using the photochemical NBT method. The samples (0.5 g) were homogenized in 5 ml extraction buffer consisting of 50 mM phosphate (pH 7.8). The assay mixture in 3 ml contained 50 mM phosphate buffer (pH 7.8), 26 mM methionine, 750 μM NBT, 1 μM EDTA, and 20 μM riboflavin. The photo reduction of NBT (formation of purple formazan) was measured at 560 nm and an inhibition curve was made against different volumes of extract. One unit of SOD is defined as being present in the volume of extract that causes inhibition of the photo reduction of NBT by 50% (Zhou et al., 1997).

### RNA-Seq-Based Transcriptome Analysis of Root and Shoot

#### RNA Extraction

The shoot and root samples before GR24 and NaCl treatment were named as N0_1 (mock 1) and N0_2 (mock 2), respectively. At 12 h after treatment, N1_1 (distilled water), N2_1 (200 mM NaCl), N3_1 (distilled water+GR24), and N4_1 (200 mM NaCl+GR24) were named for shoots, respectively. N1_2 (distilled water), N2_2 (200 mM NaCl), N3_2 (distilled water+GR24), and N4_2 (200 mM NaCl+GR24) were named for roots, respectively. The samples with three replications for each treatment were used for RNA-Seq. Total RNA was extracted using TRI reagent (MRC, Cincinnati, OH, United States) and digested with Turbo DNase enzyme (Ambion, Austin, TX, United States) as per the manufacturer’s instructions.

RNA was extracted using TRIzol according to the manufacturer’s instructions. RNA degradation and contamination were monitored on 1% agarose gels. RNA purity was checked using the NanoPhotometer^®^ Spectrophotometer (IMPLEN, CA, United States). RNA concentration was measured using Qubit^®^ RNA Assay Kit in Qubit^®^ 2.0 Flurometer (Life Technologies, CA, United States). RNA integrity was assessed using the RNA Nano 6000 Assay Kit of the Bioanalyzer 2100 System (Agilent Technologies, CA, United States).

### cDNA Synthesis, Library Construction, and Sequencing

For each of the samples within each treatment per time point, we subjected 5 μg of total RNA to one round of poly A selection on oligo(dT) Serabeads (Illumina mRNA-Seq Kits Cat no. RS-00-0801). The resultant mRNA was fragmented to an average size of 500 bp using divalent cations at 95°C for 5 min prepared following the manufacturer’s recommended protocol (Illumina mRNA-Seq Kits Cat no. RS-100-0801). First strand cDNA synthesis was carried out using Superscript III reverse transcriptase (Invitrogen) and 3 μg random hexamer primers (Illumina) per sample as per the manufactures’ instruction. Second strand cDNA synthesis and RNA-Seq samples were prepared according to the manufacturer’s recommended protocol (Illumina). The fragment size and concentration of resultant libraries were assessed on a Qubit Fluorometer (Invitrogen QuantRNA) and on a Bioanalyzer High Sensitivity Chip (Invitrogen QuantRNA). All libraries were sequenced on Hiseq2500 with 125 bp paired-end reads. The dataset has been deposited in the National Center for Biotechnology Information (NCBI; project accession number PRJNA 393467).

### Differentially Expressed Genes

We retained reads determined by software NGS QC Toolkit (Patel and Jain, 2012). These reads were aligned to the *B. napus* draft genome using SOAP (Li et al., 2008) with default settings. Only reads that mapped only once to the genome were used in the analysis. The gene expression was normalized using the value of reads per kilobase per million reads (RPKM). A recently published Bioconductor package (Gentleman et al., 2004), edgeR (Robinson et al., 2010), was also used to measure gene differential expression between the two samples. The absolute value of |log_2_ FPKM ratio|≥ 1 (under the criterion of *P*-value ≤ 0.01 and FDR ≤ 0.001) was used as threshold to assess the significance of changes in gene expression.

### GO and KEGG Analysis of DEGs

The functional annotations of the genes were determined by BLASTx against the followed database, the NCBI non-redundant (Nr) protein database and the Kyoto Encyclopedia of Genes and Genomes (KEGG) pathway database (Kanehisa, 2002) with an *E*-value cut-off of 1.0 e^-5^. On the basis of Nr annotation, the Blast2GO program (Conesa et al., 2005) was used to obtain gene ontology (GO) annotation for genes annotated by Nr. Then, the WEGO software (Ye et al., 2006) was used to perform GO functional classification for these genes. GOstat was used to identify over-represented GO category.

### Validation of Transcriptomic Analysis using qPCR

Real-time PCR was carried out using a StepOnePlus^TM^ Real-Time PCR System (Applied Biosystems) and Fast SYBR Green Master Mix (Applied Biosystems) to monitor double-stranded DNA synthesis in combination with reference dye (ROX) as a passive reference dye. PCR reactions were carried out in duplicate using 7.5 pmol specific primers and approximately 5 ng cDNA in a total volume of 15 μl. The thermal profile for amplification was as follows: 95°C for 2 min, followed by 40 cycles of 95°C for 10 s, 58°C for 30 s, and 60°C for 30 s. Primer pairs were validated by standard curve analysis, and expression levels of target genes calculated using the ΔΔ*C*_T_ method, with actin as the internal control gene. We designed primers with Primer3^1^, with the exception of the actin primer pair, which were taken from Yang et al. (2014).

### Statistical Analysis

All data presented are mean values of each treatment. Data were analyzed using the statistical program SAS, and the analysis of variance (ANOVA) was followed by Fisher’s protected LSD test to identify homogenous groups within the means. Significant differences among treatments were considered at the *P* ≤ 0.05 level.

## Results

### Fresh and Dry Weight of Plants

**FIGURE 1:**
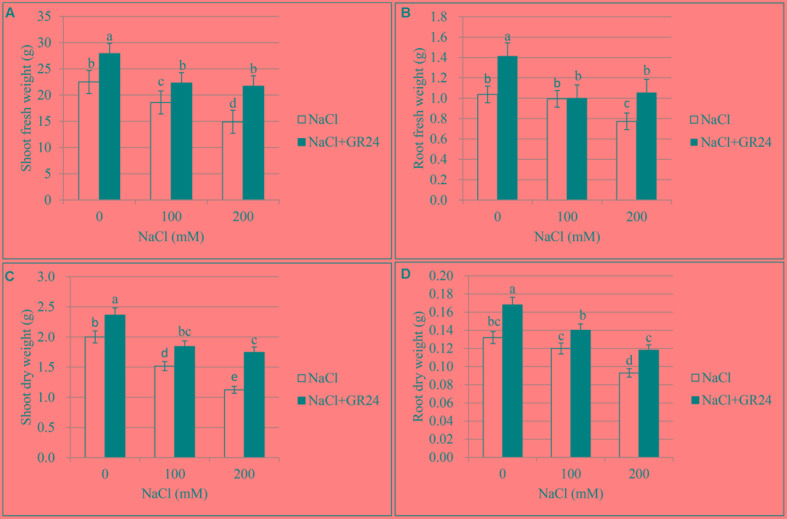
Effects of GR24 and salinity stress on the shoot (root) fresh weight, shoot (root) dry weight of seedlings in *Brassica napus* cv. ZS 11. Shoot fresh weight **(A)**, root fresh weight **(B)**, shoot dry weight **(C)**, and root dry weight **(D)** at 7 days in the presence of GR24 (0.18 μM) and salinity (0, 100, and 200 mM NaCl, respectively) in ZS 11 (*n* = 10). All experiments were repeated three times and the data represent means ± SE. Means followed by same small letters are not significantly different by the LSD test at *P* ≤ 0.05 level.

In the primary experiment, the results indicated that the root and shoot biomass increased significantly when ZS 11 was treated by GR24 with the concentration of 0.18 μM (data not shown), so 0.18 μM was selected as the optimal concentration in this experiments. The effect of three different salt contents and GR24 treatments on fresh and dry weights of plants was showed in **Figure 1**. Salt stress significantly inhibited the root and shoot growth (*P* ≤ 0.05) and both shoot fresh weight and dry weight decreased rapidly at 100 and 200 mM NaCl treatments compared to the control plants. However, GR24 treatment improved the plant growth under normal situation and helped the plants overcome salinity stress. There were no significant difference between 100 and 200 mM NaCl combined with GR24 in either shoot fresh weight or dry weight (**Figures 1A,C**). Root fresh weight and dry weight decreased significantly at 200 mM NaCl treatment, whereas there was no significant difference between control and 100 mM NaCl. Except for 100 mM NaCl treatment, GR24 increased both root fresh weight and dry weight under salinity stress (**Figures 1B,D**).

### Chlorophyll Content and Photosynthetic Characteristics

As shown in **Figure 4A**, chlorophyll content of leaves decreased significantly with increasing of salt contents. Compared with control plants, the SPAD value was decreased by 6% and 20% at 100 NaCl and 200 mM NaCl treatments, respectively. The plants under salinity stress and treated with GR24 showed significantly increased chlorophyll content. The SPAD value increased by 8 and 23%, respectively, compared to the corresponding plants under NaCl stress. The SPAD value with GR24 treatment alone was 13% higher than that of control plants.

**FIGURE 2:**
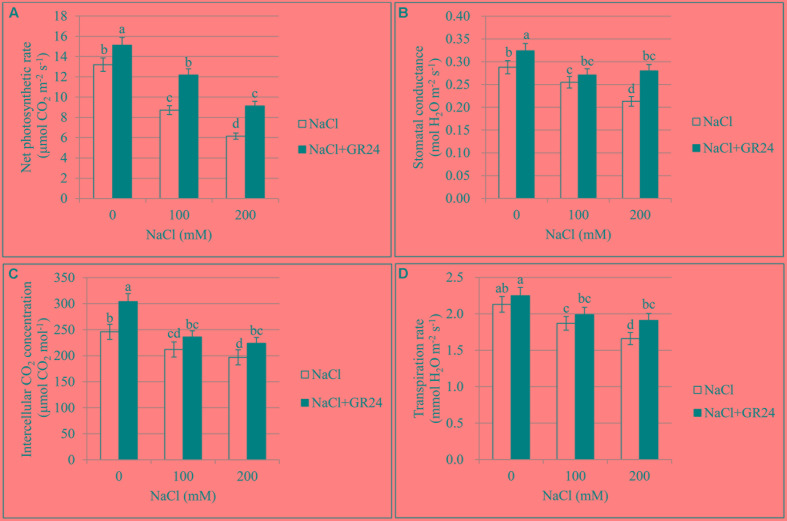
Effects of GR24 and salinity stress on the leaf photosynthetic parameters in *B. napus* cv. ZS 11. Net photosynthetic rate (Pn) **(A)**, stomatal conductance (Gs) **(B)**, intercellular CO_2_ concentration (Ci) **(C)**, and transpiration rate (Tr) **(D)** at 7 days in the presence of GR24 (0.18 μM) and salinity (0, 100, and 200 mM NaCl, respectively) in ZS 11 (*n* = 6). All experiments were repeated three times and the data represent means ± SE. Means followed by same small letters are not significantly different by the LSD test at *P* ≤ 0.05 level.

The Pn treated with 100 and 200 mM NaCl decreased significantly by 34 and 53% compared to control, respectively. However, Pn with GR24 application under salinity stress increased by 40 and 49% compared to salinity stress, respectively. Pn with GR24 treatment alone was 15% higher than that of control. The trends of Gs, Ci, and Tr were coincident with the Pn, which decreased with increasing NaCl concentrations whereas increased in combination with GR24 treatment. Comparing 100- and 200 mM NaCl-treated plants with control, Gs decreased by 12 and 26%, respectively; Ci decreased by 14 and 20%, respectively; and Tr decreased by12 and 22%, respectively. However, GR24 treatment significantly enhanced all the photosynthetic parameters (**Figure 2**).

### Chlorophyll Fluorescence

**FIGURE 3:**
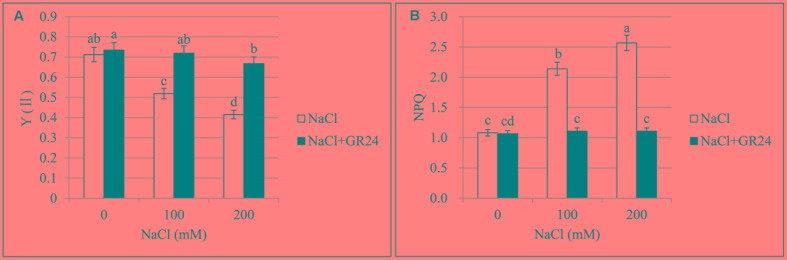
Effects of GR24 and salinity stress on the chlorophyll fluorescence from leaves in *B. napus* cv. ZS 11. Quantum yield of PSII (Y(II)) **(A)** and non-photochemical quenching (NPQ) coefficient **(B)** at 7 days in the presence of GR24 (0.18 μM) and salinity (0, 100, and 200 mM NaCl, respectively) in ZS 11 (*n* = 6). All experiments were repeated three times and the data represent means ± SE. Means followed by same small letters are not significantly different by the LSD test at *P* ≤ 0.05 level.

Photosystem II quantum yield (Y(II)) and non-photochemical quenching (NPQ) are important parameters of chlorophyll fluorescence. Y(II), the quantum yield of PSII, was decreased significantly by 27 and 42%, respectively, with the increasing of NaCl concentration compared to normal growing plants (**Figure 3A**). No significant change was observed between normal growing plants and GR24 treatment, while Y(II) increased significantly by 39 and 61%, respectively, at 100 and 200 mM NaCl in combination with GR24 treatment compared to the corresponding salinity stress. Otherwise, NPQ coefficient increased significantly by 98 and 137%, respectively, compared 100 and 200 mM NaCl treatments to normal plants (**Figure 3B**). GR24 application significantly decreased the NPQ compared with the salinity stress alone, whereas no significant difference was observed among the three treatments of GR24, GR24 in combination with 100 mM NaCl, and GR24 in combination with 200 mM NaCl.

### Lipid Peroxidation Assay and Activity of Antioxidant Enzymes

**FIGURE 4:**
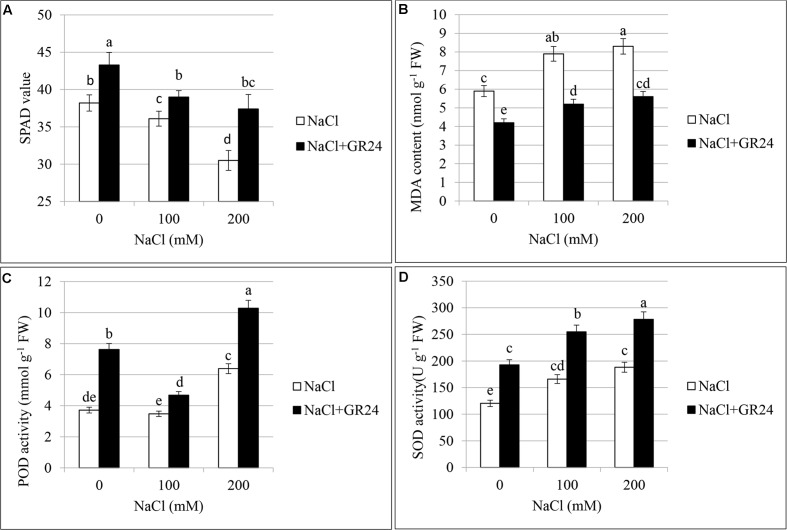
Effects of GR24 and salinity stress on the soil and plant analyzer development (SPAD) value (chlorophyll content), lipid peroxidation, and activities of antioxidant enzymes of leaves in *B. napus* cv. ZS 11. SPAD value **(A)**, malondialdehyde (MDA) contents **(B)**, guaiacol peroxidase (POD) activity **(C)**, and superoxide dismutase (SOD) activity **(D)** at 7 days in the presence of GR24 (0.18μM) and salinity (0, 100mM and 200mM NaCl, respectively) in ZS 11 (n = 6). All experiments were repeated three times and the data represent means ± SE. Means followed by same small letters are not significantly different by the LSD test at *P* ≤ 0.05 level.

**Figures 4B–D** showed the lipid peroxidation and activities of antioxidant enzymes in the leaves of *B. napus* cv. ZS 11. MDA contents increased significantly under salinity stress with the increasing of NaCl concentration (**Figure 4B**). The highest content appeared at 200 mM NaCl treatment, which increased by 81% compared to control. As expected, GR24 application decreased MDA accumulation, the contents were pronounced and significant as compared to those under corresponding salinity stress levels. Notably, MDA contents after GR24 treatment were lower than that of no-salinity-treated control.

Peroxidases increased with the increasing of NaCl contents (**Figure 4C**). There was no significant difference between normal plants and that at 100 mM NaCl treatment, whereas the POD increased significantly at 200 mM NaCl treatment compared with both normal plants and 100 mM NaCl treatment. POD increased significantly under salinity stress accompanying with GR24 application compared to the corresponding salinity stress. The value was the lowest at 100 mM NaCl in combination with GR24 treatment, whereas the highest value was observed when the plants received 200 mM NaCl and GR24 treatment in combination.

The change in trend of SOD was similar with that of POD (**Figure 4D**). There was no significant difference between 100 and 200 mM NaCl stress, while they both had significant difference with that of normal plants. POD increased significantly under salinity stress accompanying with GR24 application compared to the corresponding salinity stress. The highest value appeared when the plants received 200 mM NaCl and GR24 treatment in combination.

### Illumina Sequencing and Data Analysis

As GR24 promoted the growth of seedlings under salt stress, the physiological characters were determined and the molecular mechanism should be elucidated further. In this study, gene expression accounted for the shoot and root growth was analyzed. Root and shoot samples from ZS 11 under salt stress, salt stress with GR24 treatment, and control conditions were used to construct 10 libraries for sequencing. About 5.3 million of raw reads and 5 million of clean data for each library were obtained, respectively, and 68% of the clean data could be mapped (**Table 1**). Among which, the multiple mapped reads were approximately 2 million and accounted for 4% of total mapped reads. The uniquely mapped reads were approximately 3 million and accounted for 65% of total mapped reads.

**Table 1:** The data from sequencing different samples.

Sample name	N0_1	N0_2	N1_1	N1_2	N2_1	N2_2	N3_1	N3_2	N4_1	N4_2
Raw reads	54880134	60041638	58411446	45682780	50409982	54074866	52483466	53296894	46666958	49954454
Clean reads	51830048	56468926	54973534	43057428	47374290	50733762	49467890	50256354	43987424	47027946
Total mapped	37002173	37215139	38800197	28361177	33444807	32981411	35298536	32324309	31446421	31700111
	(71.39%)	(65.9%)	(70.58%)	(65.87%)	(70.6%)	(65.01%)	(71.36%)	(64.32%)	(71.49%)	(67.41%)
Multiple mapped	1939666	1893865	2329650	1481590	1939819	1733252	1748976	1738803
	(3.74%)	(3.35%)	(4.24%)	(3.44%)	(4.09%)	(3.42%)	(3.54%)	(3.46%)	1732094 (3.94%)	1377128 (2.93%)
Uniquely mapped	35062507	35321274	36470547	26879587	31504988	31248159	33549560	30585506	29714327	30322983
	(67.65%)	(62.55%)	(66.34%)	(62.43%)	(66.5%)	(61.59%)	(67.82%)	(60.86%)	(67.55%)	(64.48%)


**FIGURE 5:**
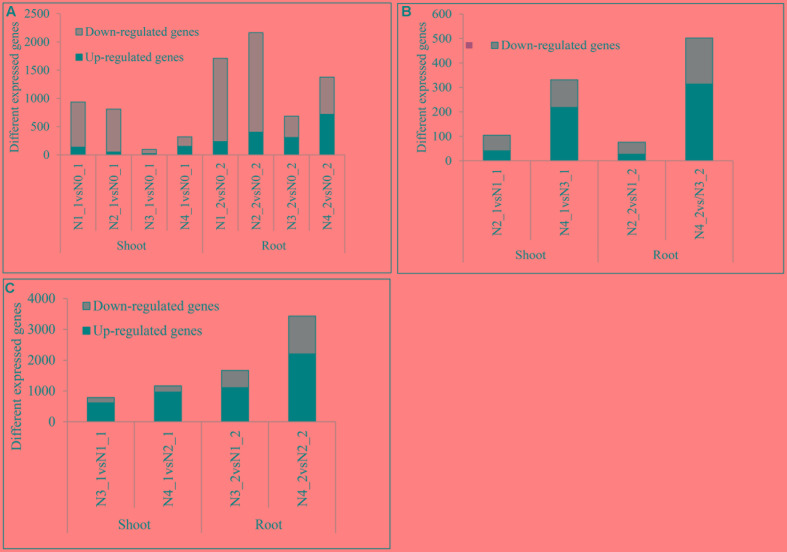
Summaries of differentially expressed genes (DEGs) at salinity and GR24 treatments. DEGs were identified by comparing each treatment to N0 **(A)**, DEGs under salinity stress **(B)**, and DEGs under GR24 treatment **(C)**. The “N1_1 vs N0_1” indicates a comparison between the gene expression in the N1_1 library with that in the N0_1 library, and the same describes the other labels on the *x*-axis. DEGs were confirmed based on whether the |log_2_ FPKM ratio|≥ 1 and *P*-value ≤ 0.01.

Compared with the pre-treated sample, there were 937, 810, 95, and 320 DEGs at distilled water, NaCl, distilled water in combined with GR24, and NaCl in combined with GR24-treated shoots, respectively. Correspondingly, there were 1710, 2163, 686, and 1376 DEGs at distilled water, NaCl, distilled water in combined with GR24, and NaCl in combined with GR24-treated roots, respectively. Interestingly, the down-regulated genes outnumbered the up-regulated genes in NaCl combined with GR24-treated roots. It was notably that GR24 treatment reduced the number of DEGs significantly either in shoot or root (**Figure 5A**).

The DEGs that respond to the salinity were identified (**Figure 5B**). The DEGs under NaCl in combined with GR24 vs distilled water in combined with GR24 treatments were 331 and 502 in shoot and root, respectively, and the up-regulated outnumbered the down-regulated genes, which were much more than that at NaCl vs distilled water treatments with 105 and 76 DEGs in shoot and root, respectively, and the down-regulated outnumbered the up-regulated genes. The results mean GR24 promoted the gene expression of salinity stress.

The effects of GR24 treatment under distilled water and salinity background were identified (**Figure 5C**). In N3_1 vs N1_1, N4_1 vs N2_1, N3_2 vs N1_2, and N4_2 vs N2_2, 615, 964, 1106, and 2205 genes were up-regulated and 173, 199, 560, and 1228 genes were down-regulated, respectively. The up-regulated genes were much more than the numbers of down-regulated ones. Moreover, the numbers of DEGs after GR24 treatment were more in the root than in the shoot as well as under the salinity stress than treated with the distilled water. The results mean salinity promoted the gene expression of GR24 treatment.

**FIGURE 6:**
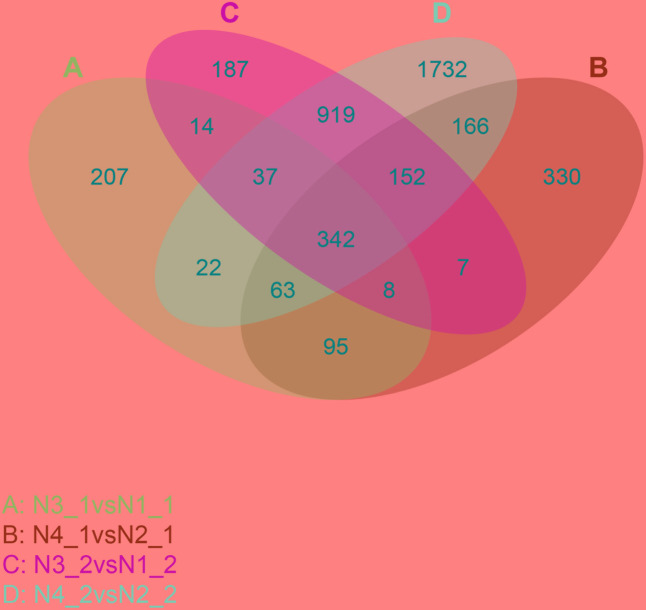
Venn diagram describing the exclusion and overlap of regulated genes after GR24 treatment under salinity stress. The “N3_1 vs N1_1” **(A)** indicates a comparison between the gene expression in the N3_1 library with that in the N1_1 library, and the same describes the other labels **(B–D)**.

From the DEGs analysis, we found that gene expression changed greatly after GR24 treatment, so more attention was given to identify these genes. The Venn diagram showed that about 508 genes commonly expressed under the distilled water and salinity background in the shoot, while 655 genes were unique expressed under salinity background. Meanwhile, about 1450 genes commonly expressed under the distilled water and salinity background in the root, while 1983 genes were uniquely expressed under salinity stress. Three hundred and forty-two genes were differentially expressed under GR24 treatments. When N4_1 vs N2_1 and N4_2 vs N2_2 were analyzed, only166 genes were specially identified in shoot and root under salinity stress after GR24 treatment (**Figure 6**).

### Expressed Patterns of Candidate Genes Related to GR24 Treatment

**FIGURE 7:**
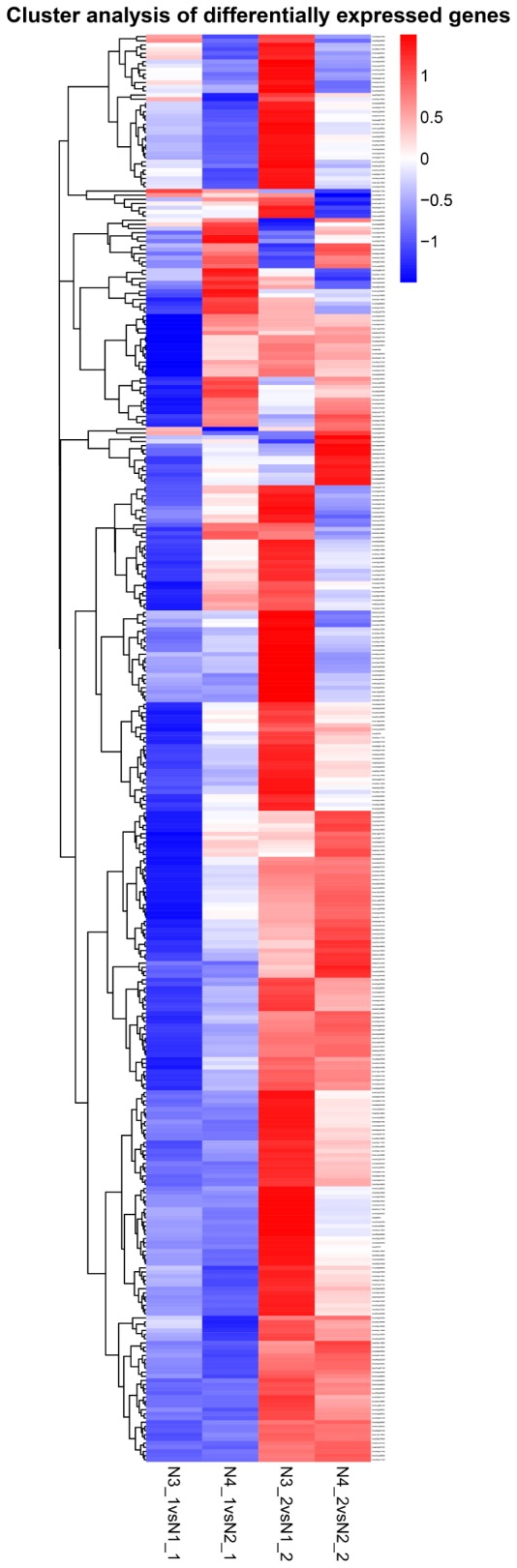
Hierarchical cluster analyses of 342 common DEGs after GR24 treatment. This map shows the genes of log_2_ (foldchange) values of the GR24 effects on shoot and root. The “N3_1 vs N1_1” indicates a comparison between the gene expression in the N3_1 library with that in the N1_1 library, and the same describes the other labels.

**FIGURE 8:**
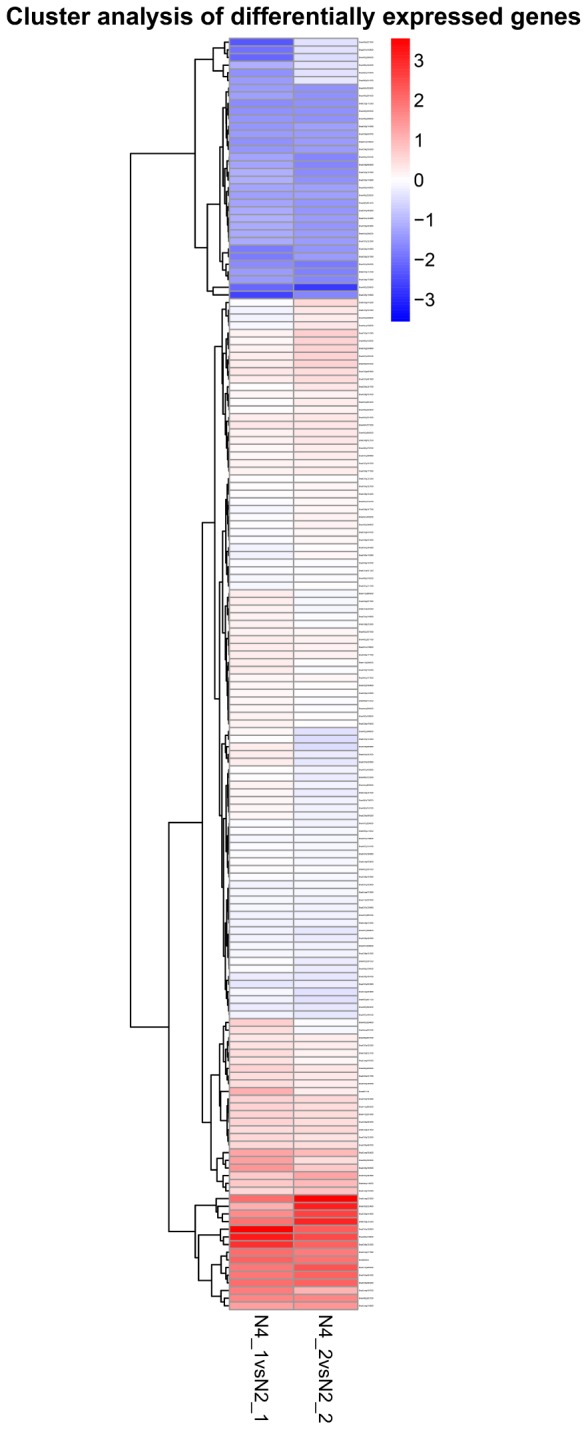
Hierarchical cluster analyses of 166 special DEGs after GR24 treatment under salinity stress. This map shows the genes of log_2_ (foldchange) values of GR24 effects on shoot and root. The “N4_1 vs N2_1” indicates a comparison between the gene expression in the N4_1 library with that in the N2_1 library, and the same describes as “N4_2 vs N2_2.”

By comparing the gene expression levels between the distilled water and salt stress, the analysis to the effects of GR24 treatment in shoot and root showed complicated dynamic expression patterns for 342 common differentially expressed genes (DEGs) (**Figure 7**), but normally seven clusters were classified, more down-regulated DEGs were expressed in shoot after GR24 treatment. Nevertheless, more up-regulated DEGs were expressed in root. The cluster in the bottom is ranked as the most stable, 35 genes were down-regulated in shoot as well as up-regulated in root. We also analyzed 166 DEGs after GR24 treatment only under salt stress in shoot and root. Totally, three clusters were appeared. In the top cluster, 34 genes were down-regulated in both shoot and root, in the bottom cluster, 15 genes were up-regulated (**Figure 8** and **Supplementary Table S1**).

### GO and KEGG Analysis for DEGs

**FIGURE 9:**
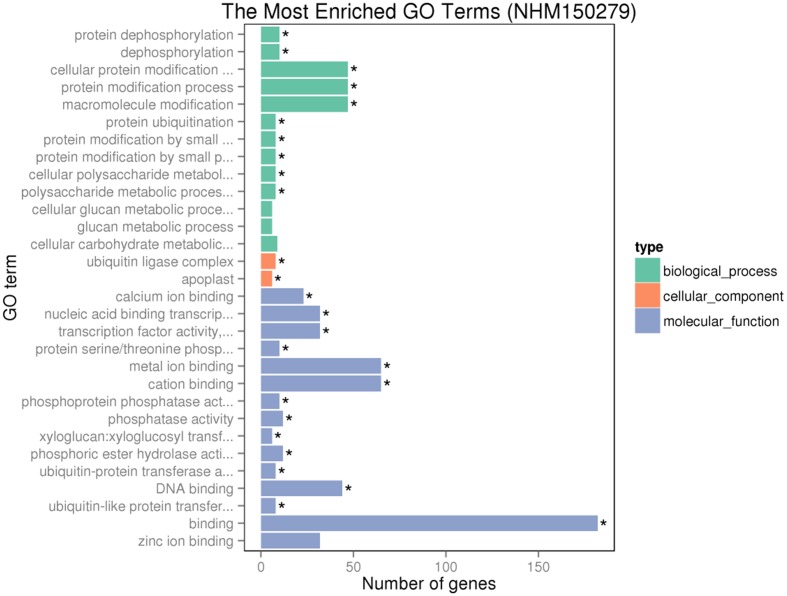
Gene ontology (GO) analyses of the 342 common DEGs after GR24 treatment. The DEGs were assigned into three groups with different colors, including biological process (BP), cellular components (CCs), and molecular function (MF). The *x*-axis represents the number of genes in each category, and *y*-axis represents the most abundant categories in each group.

**FIGURE 10:**
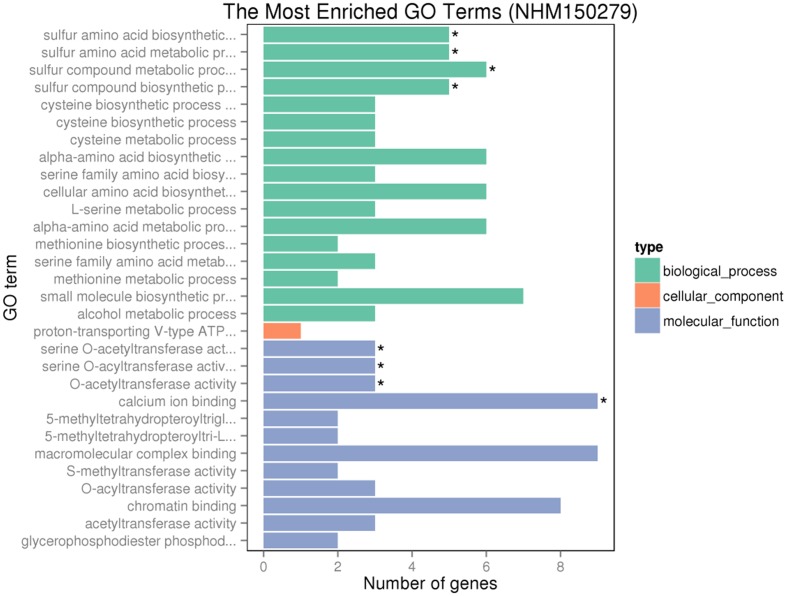
GO analyses of 166 special DEGs after GR24 treatment under salinity stress. The DEGs were assigned into three groups with different colors, including BP, CCs, and MF. The *x*-axis represents the number of genes in each category, and *y*-axis represents the most abundant categories in each group.

Gene ontology assignments for the function of 342 and 166 genes were classified. Three GO categories of 342 genes were presented in **Figure 9**. In the biological process (BP) category, the most abundant GO terms were “cellular protein modification process,” “protein modification process,” and “macromolecule modification process.” In the cellular component (CC) category, “ubiquitin ligase complex” was the most abundant, followed by “apoplast.” Similarly, in the molecular function (MF) category, “binding” was the most abundant category, followed by “metal ion binding,” “cation binding,” and “DNA binding.” In **Figure 10**, in the BP category, “sulfur compound metabolic process” was the most abundant, followed by “sulfur amino acid biosynthetic process,” “sulfur amino acid metabolic process,” and “sulfur compound biosynthetic process.” In the CC category, “proton-transporting V-type ATP” was the most abundant. In the MF category, “calcium ion binding” was ranked the most abundant term, followed by “macromolecular complex binding” and “chromatin binding.”

In **Figure 7**, we could find 35 genes down-regulated in shoot while up-regulated in root. A total of six genes could be aligned to the KEGG pathways (**Supplementary Table S2**). The pathways with more mapped genes were the plant hormone signal transduction (*BnaC02g11480D*, *BnaA10g11500D*); glycolysis/gluconeogenesis (*BnaC01g40210D*); biosynthesis of secondary metabolism (*BnaC09g24050D*). In **Figure 8**, 34 down-regulated and 15 up-regulated genes were observed in both shoot and root. A total of 16 genes and 4 genes could be aligned to the KEGG pathways (**Supplementary Table S2**), respectively. The pathways with more mapped genes were mainly the tryptophan metabolism (*BnaA09g52790D*, *BnaA01g13280D*); plant hormone signal transduction (*BnaA03g38630D*, *BnaA01g33420*); biosynthesis of amino acids (*BnaC04g05620D*, *BnaC03g33530D*, *BnaA08g00140D*, *BnaA03g28400D*, *BnaC05g48620D*); oxidative phosphorylation (*BnaA08g21880D*); plant–pathogen interaction (*BnaC01g38680D*). In N4_1 vs N2_1 DEG enriched KEGG pathway, the genes related to photosynthesis (*BnaC08g44890*) and photosynthesis–antenna proteins (*BnaA08g17660*, *BnaA07g07560D*) were mapped (**Supplementary Table S2** and **Figure S1**).

### DGE and qPCR Verification

**FIGURE 11:**
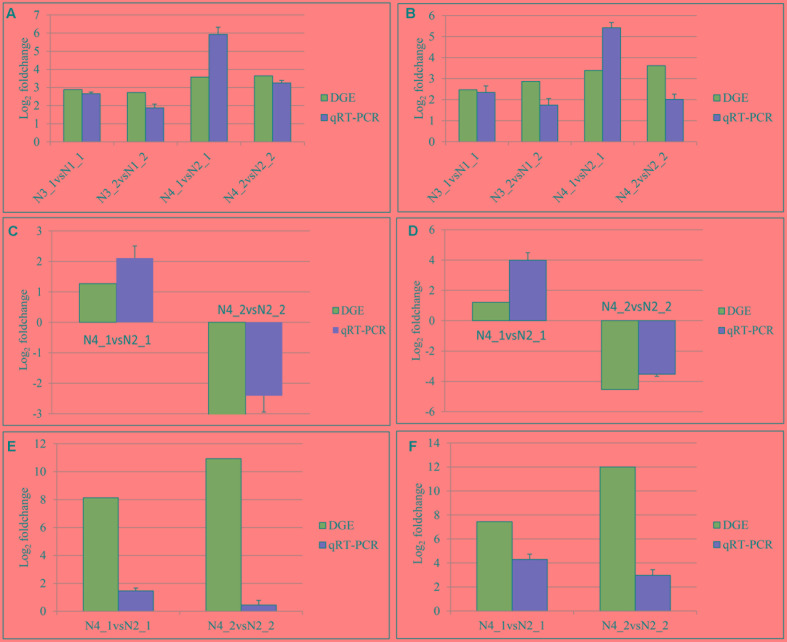
Quantitative real-time PCR (qPCR) validations of gene expression levels from digital gene expression (DGE) analysis. The qPCR values are presented as the averages of three independent experiments. The genes were randomly selected. “N3_1 vs N1_1” indicates a comparison between the gene expression in the N3_1 library with that in the N1_1 library, and the same describes the other labels. The *y*-axis indicates the log_2_ (foldchange) obtained by two methods. The **(A–F)** indicates the genes with the assay names of *BnaA10g11500D*, *BnaC02g11480D*, *BanA01g13280D*, *BanC08g44890D*, *BanA08g21880D*, and *BnaC01g38680D*, respectively.

A total of 24 genes mapped as tryptophan metabolism, plant hormone signal transduction, and photosynthesis in KEGG pathway were evaluated by qPCR (**Supplementary Table S3**). The data of qPCR were in **Supplementary Figure S2**. The comparisons of the relative expression levels of six genes were shown in **Figure 11**. The results showed that the similar expression patterns were consistent with the DGE data.

## Discussion

Plants’ survival depends largely on the ability of these sessile organisms to sense environmental changes, integrate external signals with their own developmental programs, and produce appropriate responses (Stepanova et al., 2005). Root is the first organ of plant affected by salinity and it influences ion accumulation and shoot growth (Hu et al., 2016). In view of the results presented herein, it is quite clear that salinity stress at the seedling stage resulted in a significant decline in root and shoot biomass accumulation. As noted, when GR24 was applied to treat the roots, it could move from roots into shoots (Koltai, 2011; Khosla and Nelson, 2016), and played a positive role in plant responses to the salinity stress (Ha et al., 2014; Kapulnik and Koltai, 2014). All the morphological, physiological, and biochemical responses to GR24 application in our experiments support the idea that a strong root–shoot interaction exists in rapeseed.

Leaves are the main site of Na^+^ toxicity for most plants (Munns and Tester, 2008). MDA is served as an indicator of the extent oxidative damage in stressed plants. The results reported here showed that the degree of accumulation of MDA was higher due to salinity stress, indicating a high rate of lipid peroxidation in rapeseed. However, although the MDA increased significantly either at 100 or 200 mM NaCl treatment compared to the control, there was no significant difference between these two treatments. As reported, rapeseed is moderately tolerant to salinity (Ashraf and McNeilly, 2004), so it is speculated that the polyunsaturated fatty acids in the membrane undergo peroxidation reach a relative balance at 100 and 200 mM NaCl stress. Most importantly, MDA reduced significantly after GR24 treatment and there was no significant difference between 100 mM NaCl in combination with GR24 and 200 mM NaCl in combination with GR24 treatments, which demonstrated the strong positive effect of GR24 on preventing the membrane from stress damage. The similar phenomenon appeared on the SPAD values, which meant a less chloroplast damage after GR24 treatment. In addition, salinity can significantly accelerate the generation of ROS at cellular level (Gill and Tuteja, 2010; Rasool et al., 2013). Radical forms of ROS majorly include superoxide radicals ($O_{2}^{•-}$), perhydroxy radical (${\mathrm{HO}}_{2}^{•}$), and alkoxy radicals (RO), whereas hydrogen peroxide (H_2_O_2_) and singlet oxygen (^1^O_2_) are included in non-radical molecular form (Nath et al., 2016). SOD is a major scavenger of superoxide ($O_{2}^{•-}$), and its enzymatic action results in the formation of H_2_O_2_ and ^1^O_2_. POD decomposes H_2_O_2_ by oxidation of co-substrates such as phenolic compounds and/or antioxidants (Meloni et al., 2003; Wang et al., 2014). The significant increases in SOD and POD activities suggest GR24 has a positive effect on scavenging the ROS generated by salinity stress in rapeseed.

Salinity also affects the cell organelle like chloroplast and it is the site for most of photosynthetic processes (PSI and PSII) (Nusrat et al., 2014). The inhibition of photosynthesis under salinity stress may be partially attributed to the stomatal closure (Steduto et al., 2000). Our results suggest the reduction in Gs accompanied by decreased leaf chlorophyll content could have contributed to the decrease of photosynthesis, which is consistent with reports by Naeem et al. (2012). However, the exogenous application of GR24 significantly alleviated the inhibiting effects of salinity stress, which was evidenced by an increase in the photosynthetic capacity.

Chlorophyll fluorescence imaging has been successfully applied for the detection of biotic or abiotic stresses in plants (Gorbe and Calatayud, 2012) as a diagnostic tool. In this study, the Y(II) decreased in the salinity stressed leaves, which demonstrated the decrease of absorbed quanta which converted into chemically fixed energy by the photochemical charge separation at PSII reaction centers and increase of quanta which dissipated into heat and fluorescence. The NPQ parameter is a measure of non-photochemical quenching, reflecting down-regulation of PSII as a protective mechanism against excess light intensity (Kramer et al., 2004). A low NPQ value after GR24 treatment indicated the samples alleviated the photosynthetic processes by dissipation of excessive excitation energy into harmless heat.

Much progress has been made to unravel the effects of SLs on the root growth and shoot branching of various plant species (Koltai et al., 2010; Brewer et al., 2013; Foo and Reid, 2013; Matthys et al., 2016). The present findings indicate that GR24 improves the root–shoot growth and physiological processes under salinity stress. Moreover, transcriptome sequencing is a proven strategy for expression profiling of genes involved in various processes in plants (Pan et al., 2014). Through expression comparison, GO and KEGG enrichment analyses, some genes and related pathways were identified, including “sulfur compound metabolic process,” “calcium ion binding,” and so on (**Figure 10**). S-compounds such as cysteine (Cys), glutathione (GSH), and glucosinolates (GSs) directly or indirectly modulated/regulated by ATP-sulfurylase are involved in plant tolerance to salinity stress, i.e., ROS scavenging and cellular redox homeostasis (Anjum et al., 2015). Calcium, serving as a common second messenger for abiotic stress, reduces the accumulation of Na (+), H_2_O_2_, and MDA (Wang et al., 2013). It is noteworthy that exogenous GR24 induces the expression of genes aligned to the KEGG pathways of photosynthesis, carbon metabolism, and photosynthesis–antenna proteins.

Phytohormones are known to interact with each other to regulate specific phenotypes and adapt environmental stress, including the abscisic acid (ABA), cytokinin, auxin, and strigolactone pathways (Ha et al., 2014; Khosla and Nelson, 2016). It is reported that cross-talk between SLs and ABA plays an important role in integrating stress signals to regulate stomatal development and function (Ha et al., 2014). In other literatures, the tight cross-talk between SLs and IAA affecting shoot and root development has been highlighted (Crawford et al., 2010; Domagalska and Leyser, 2011; Shinohara et al., 2013). Tryptophan is the precursor of IAA. In the present work, KEGG pathways of tryptophan metabolism and plant hormone signal transduction were detected after GR24 treatment under saline conditions. Thus, we suppose that the beneficial effects of GR24 in the response to salinity have been also associated to a cross-talk with IAA in rapeseed.

## Author Contributions

NM: conceived and designed the experiments. NM and CH: performed the experiments. NM and LW: analyzed the data. QH and JX: contributed reagents/materials/analysis tools. NM: wrote the manuscript. NM and CZ: revised the manuscript. All the authors showed the final approval of the version to be published. All authors agreed to be accountable for the content of the work.

## Conflict of Interest Statement

The authors declare that the research was conducted in the absence of any commercial or financial relationships that could be construed as a potential conflict of interest.

## Footnotes

1 http://primer3.ut.ee/

## Supplementary Material

**TABLE S1 |** The description for 35 genes commonly down-regulated in shoot and up-regulated in root after GR24 treatment, 34 genes specially down-regulated in shoot and root, and 15 genes specially up-regulated in shoot and root after GR24 treatment under salinity stress.

**TABLE S2 |** Genes aligned to KEGG pathway.

**TABLE S3 |** The genes evaluated by qPCR.

**FIGURE S1 |** Statistics of pathway enrichment.

**FIGURE S2 |** The relative expressed level for 24 genes.
