# Supplementary figures and images for: Strigolactones Improve Plant Growth, Photosynthesis, and Alleviate Oxidative Stress under Salinity in Rapeseed (Brassica napus L.) by Regulating Gene Expression
Source: Front Plant Sci. 2017 Sep 27;8:1671. doi: 10.3389/fpls.2017.01671 (PMC5623956; doi:10.3389/fpls.2017.01671)

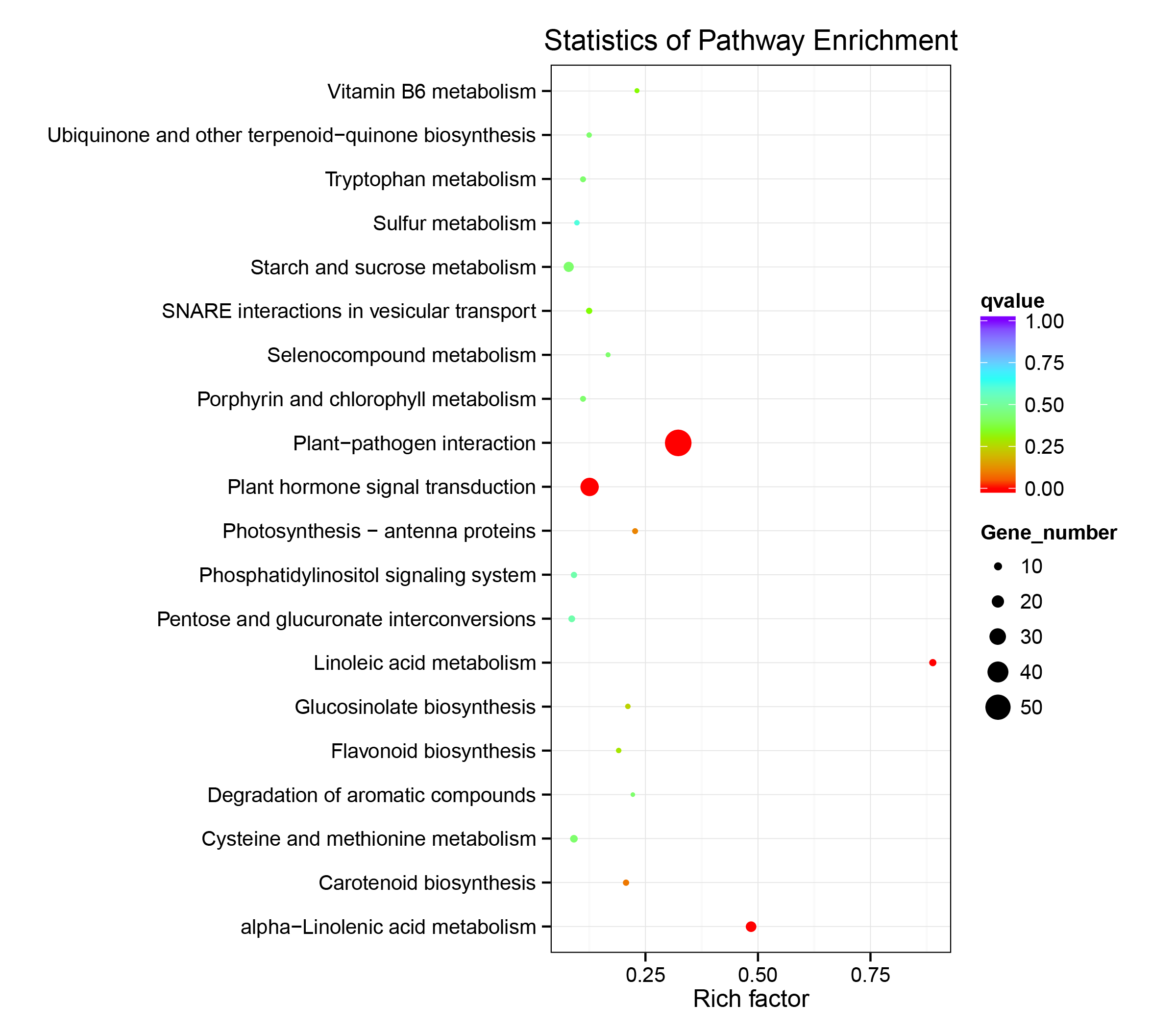

Supplement: Supplementary file 4 [file Image_1.TIF]

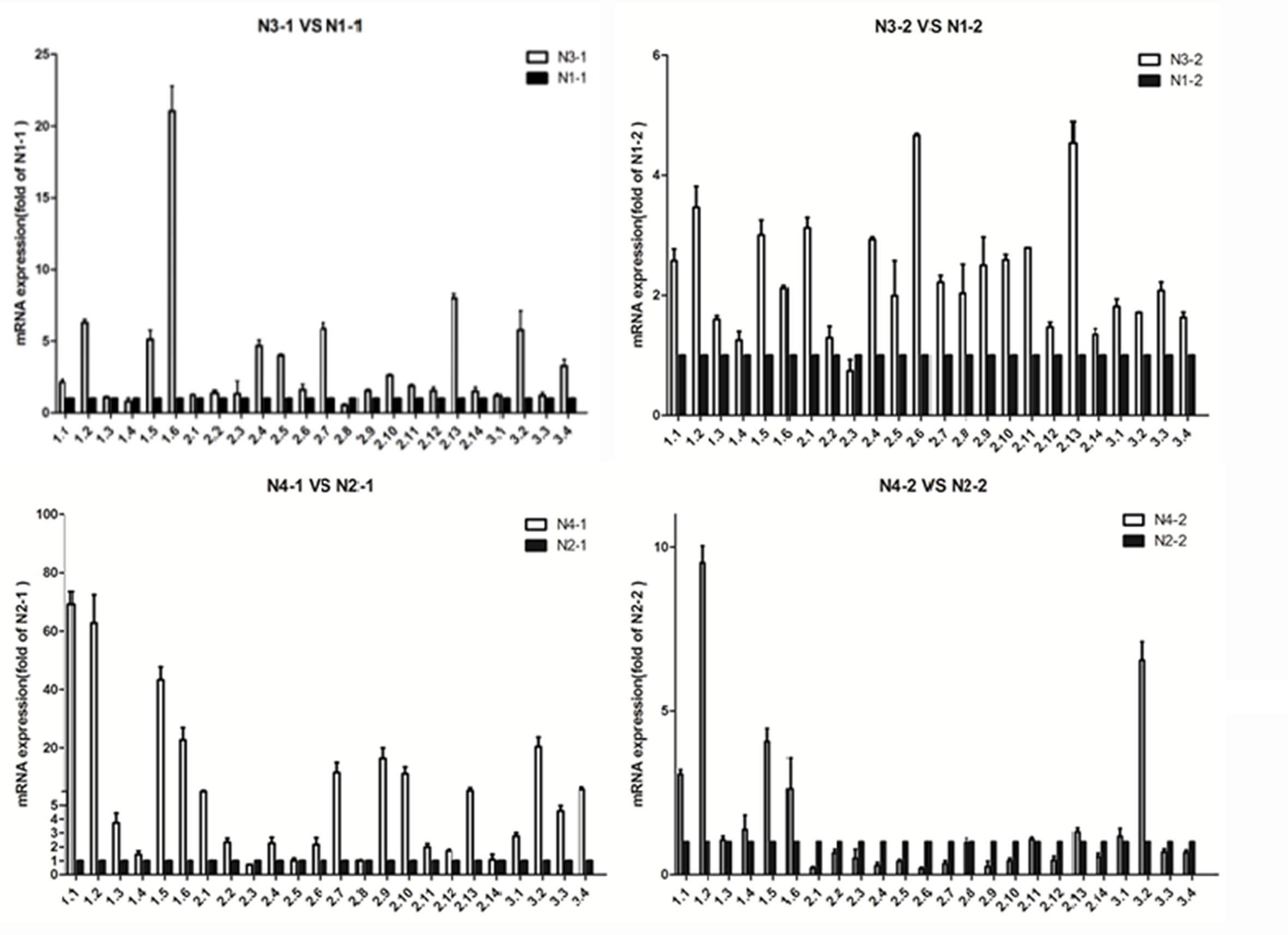

Supplement: Supplementary file 5 [file Image_2.TIF]
